# Differences in *In Vitro* Disintegration Time among Canadian Brand and Generic Bisphosphonates

**DOI:** 10.1155/2014/420451

**Published:** 2014-10-02

**Authors:** Wojciech P. Olszynski, Jonathan D. Adachi, K. Shawn Davison

**Affiliations:** ^1^Saskatoon Osteoporosis Centre and Department of Medicine, University of Saskatchewan, Suite 103, 39-23rd Street East, Saskatoon, SK, Canada S7K 0H6; ^2^Department of Medicine, St. Joseph's Hospital, McMaster University, 501-25 Charlton Avenue E., Hamilton, ON, Canada L8N 1Y2; ^3^University of Victoria, P.O. Box 1700 STN CSC, Victoria, BC, Canada V8W 2Y2

## Abstract

The objective of this study was to compare the disintegration times among Canadian-marketed brand (alendronate 70 mg, alendronate 70 mg plus vitamin D 5600 IU, and risedronate 35 mg) and generic (Novo-alendronate 70 mg and Apo-alendronate 70 mg) once-weekly dosed bisphosphonates. All disintegration tests were performed with a Vanderkamp Disintegration Tester. Disintegration was deemed to have occurred when no residue of the tablet, except fragments of insoluble coating or capsule shell, was visible. Eighteen to 20 samples were tested for each bisphosphonate group. The mean (±standard deviation) disintegration times were significantly (*P* < 0.05) faster for Apo-alendronate (26 ± 5.6 seconds) and Novo-alendronate (13 ± 1.1 seconds) as compared to brand alendronate (147 ± 50.5 seconds), brand alendronate plus vitamin D (378 ± 60.5 seconds), or brand risedronate (101 ± 20.6 seconds). The significantly faster disintegration of the generic tablets as compared to the brand bisphosphonates may have concerning safety and effectiveness implications for patients administering these therapies.

## 1. Introduction

Three-year randomized, controlled phase III clinical trials have established that the brand amino-bisphosphonates alendronate [1, 2], risedronate [3, 4], and zoledronic acid [5, 6] are generally safe and provide significant antifracture efficacy at vertebral, nonvertebral, and hip sites. Consequently, the amino-bisphosphonates are considered first-line therapy for the treatment of individuals at high risk for fracture [7].

In phase III clinical trials, therapies are assessed for two primary outcomes: efficacy and safety. Phase III trials for brand antifracture medications require large samples (>5000 patients), due to the relative low rate of fragility fracture, and a lengthy duration of observation (at least three years), in order to demonstrate efficacy and long-term safety. Contrastingly, approval for the production, marketing, and sale of generic versions of brand bisphosphonates do not require similar investigations [8].

In Canada, generic versions of a brand drug are adjudicated for approval by Health Canada, which takes into consideration the active ingredient, product strength, disease indication, route of administration, quality, and bioequivalence in comparison to the innovator product [9]. Bioequivalence of generic and brand products is assumed when dissolution and short-term bioavailability studies are completed for the generic and are found to be within an acceptable range as compared with the innovator product (90% confidence interval of the ratio of the mean area under the curve and peak concentration values of the test to reference product should be between 80% and 125%) [8, 10]. Bioequivalence studies are of a crossover design and completed with a relatively small number of young (aged 18–55 years), healthy adult volunteers of normal body weight; it is assumed that bioequivalence findings from young adults will be similar to what would be observed in significantly older patients for whom osteoporosis drugs are intended, many of whom have difficulty swallowing and orienting pills in the back of their mouths [11].

While the potency and composition of the active drug in generic versions of alendronate must be similar to brand alendronate, differences are permitted in their formulation and inactive ingredients (excipients) as long as the proportion of active drug to excipient is constant between the brand and generic forms [10]. When brand alendronate was first marketed, there were numerous postmarketing adverse event reports of esophagitis which promoted the manufacturer to redesign the tablet into a waxed, polished tablet to better allow rapid swallowing with a low change of esophageal adhesion. Further, strict dosing instructions were provided on the drug packaging to maximize the probability of the alendronate tablet reaching the gut undisintegrated and to have ample time for absorption before the introduction of food or drink (aside from water).

In Canada, generic alendronate (70 mg/week) has been available since July 2005 and recently, generic risedronate (35 mg/week) has become available as well.

Markedly different tablet disintegration times among the generic forms of alendronate and brand alendronate have been previously reported from around the world [11–14]. Disintegration is a physical process whereby a tablet is broken down into a soft mass of fine particles. Comparatively, dissolution is a measure of the rate at which the active ingredient dissolves into liquid (i.e.,* in vitro* water,* in vivo* water, and stomach acid). Disintegration must occur prior to dissolution.

The bioavailability of alendronate is exceedingly low (0.76%; 95% confidence interval 0.58–0.98) [15] and any factors that increase the probability of it being bound to food or drink further impair the absorption of alendronate, significantly limiting its effectiveness. If the disintegration time of generic alendronate was significantly slower than brand alendronate, then it would be possible for the drug to be bound and made inert by food and drink consumed after ingestion of the drug. Conversely, if the disintegration time of generic alendronate was significantly faster than that of brand alendronate, then the generic version would make alendronate available earlier and increase the risk of the active drug coming into contact with esophageal mucosa, thereby increasing the risk for oesophageal adverse events [16–18]. Further, fast-disintegrating versions of alendronate tablets may potentially decrease effectiveness as alendronate bound to the mucosa could have a greater probability of coming into contact with subsequently swallowed food or drink. Thus, significant differences in alendronate tablet disintegration time from brand alendronate may have an impact on generic alendronate and/or safety.

To date, no investigation has directly compared the disintegration times of Canadian-marketed generic alendronate tablets with those of brand alendronate and risedronate. This investigation compared the disintegration times of two versions of generic alendronate available in Canada with those of brand risedronate and two forms of brand alendronate (with or without supplementary vitamin D).

## 2. Materials and Methods

This investigation assessed the time to disintegration of brand alendronate (70 mg), brand alendronate (70 mg) with added vitamin D (5600 IU), brand risedronate (35 mg), and two forms of generic alendronate available in Canada (Apo-alendronate 70 mg: Apotex Inc., Toronto, Canada, and Novo-alendronate 70 mg: Novopharm Ltd., Toronto, Canada). These generic versions were selected for testing as they are the most widely available generic forms in Canada. All tablets were obtained from a retail pharmacy in Canada and remained unopened until testing.

A Vanderkamp Disintegration Tester (Model 71A 1013-3/10-1014) from Van Kel Industries Inc. (Cary, NC, USA) was employed for all disintegration tests. Disintegration times were measured visually* in vitro* as per standard United States Pharmacopeia disintegration method [19] in United States Pharmacopeia water at 37°C using a basket-rack assembly. One tablet was placed in each of the six tubes of the basket-rack assembly, after which the apparatus was started. Disintegration was deemed to have occurred when no residue of the tablet, except fragments of insoluble coating or capsule shell, were visible. The time to tablet disintegration was recorded for each test tube. All assessments were performed in the Department of Chemistry, University of Saskatchewan (Saskatoon, SK, Canada).

Basic descriptive statistics were completed for all tablet groups. Analysis of variance was used to determine whether significant differences existed in disintegration time among the tablets tested and the Tukey studentized range test for significant differences between specific tablet groups. Alpha was set at *P* < 0.05 and all procedures were performed with SAS 9.1.3 (SAS, Cary, NC, USA).

## 3. Results

**Figure 1:**
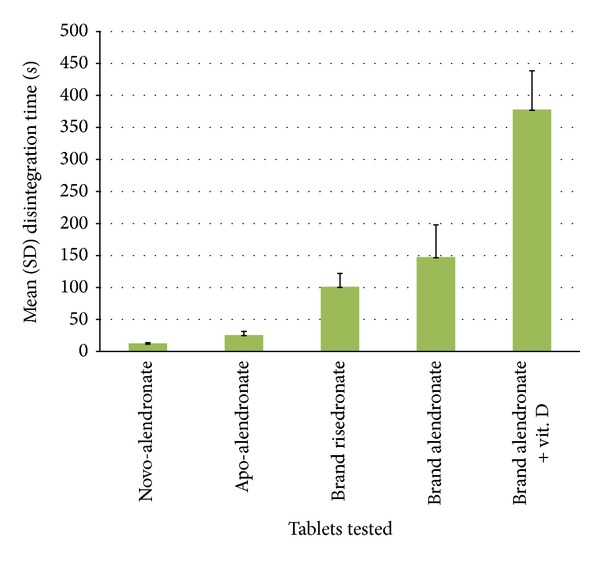
Mean (standard deviation) amino-bisphosphonate tablet disintegration times.

The lot number, sample size, and mean disintegration times for all tablets are presented in Table 1 and graphically displayed in Figure 1.

Results of the Tukey analyses are presented in Table 2. There was no significant difference in mean disintegration time between the two generic forms of alendronate, but the generic forms of alendronate had a significantly (*P* < 0.05) faster mean disintegration time than all brand formulations (alendronate, with or without vitamin D, or risedronate). Brand alendronate with vitamin D had a significantly (*P* < 0.05) slower mean disintegration time than all other tablets tested.

## 4. Discussion

This investigation found that two generic versions of alendronate available in Canada had significantly faster disintegration times than brand alendronate or brand risedronate. If generic alendronate tablets are disintegrating* in vivo* as rapidly as observed in this investigation* in vitro,* then the probability of alendronate coming into contact with the esophageal mucosa is enhanced, increasing the probability of esophageal adverse events. The disintegration time of brand alendronate plus vitamin D had not been assessed in a published trial previously and the significant (*P* < 0.05) difference in the mean disintegration time between the brand alendronate and brand alendronate plus vitamin D was notable. The longer disintegration time may result in lower absorption as compared to the other brand bisphosphonates, but if administered correctly with respect to food and drink guidelines it would be unlikely to come in contact with food or drink before adequate drug was absorbed. Assuming equal potency and composition of alendronate, the manufacturing and excipient differences may explain the significant differences in disintegration times between brand and generic alendronate observed in this and other studies [11–14].

Rapidly disintegrating generic alendronate tablets may possess a different tolerability profile as compared to the well-described safety profile of brand alendronate [20], particularly for adverse events of the esophagus. If a tablet were to prematurely disintegrate in the mouth or esophagus, alendronate becomes exposed to the oral and esophageal tissues, increasing irritation risk [21]. The significantly faster disintegration observed with the generic alendronate tablets tested in this study in concert with a slower, age-related impairment of oesophageal peristalsis may increase the risk for adverse events relating to increased esophageal drug exposure. Further, any alendronate that became bound to the esophagus would have a greater probability of being made inert and ineffective by subsequently ingested food or drink before absorption.

Epstein et al. [12] reported that some generic versions of alendronate (70 mg) available in Latin and South America disintegrated faster than brand alendronate (mean disintegration times 6.9–46.5 seconds) and some disintegrated far slower than brand alendronate (10.3–46.5 minutes). The biologic consequence of this longer disintegration time is unknown. Dansereau et al. [11] evaluated the* in vitro* disintegration and dissolution of 70 mg alendronate tablets available in Canada, Germany, The Netherlands, and the United Kingdom compared to brand alendronate. The authors also assessed disintegration rates of commercially available (nonbisphosphonate) orally disintegrating tablets, designed to disintegrate on the tongue without water, to act as a comparator group. Six of the 26 generic forms of alendronate examined had disintegration times similar to the orally disintegrating tablets (<30 seconds). The authors concluded that despite the dissolution profiles of all tablets examined being within the acceptable United States Pharmacopeia 30 specifications, the rapid disintegration of some of the copies may result in increased drug exposure in the mouth and/or esophagus [11]. A subsequent study of United States-marketed versions of generic alendronate reported that disintegration times of generic alendronate were also within the limits regulated for orally disintegrating tablets (<30 seconds) [13]. Of note is that both Canadian generics tested here would fall under that category of tablets engineered to rapidly disintegrate in the mouth (<30 seconds) and would therefore likely have an increased exposure to the oral and esophageal tissues as compared to the slower disintegrating brand bisphosphonates tested. Walker and Adachi [22] found substantial differences between brand and generic versions of risedronate marketed in Canada, although the full disintegration times were longer for all the generic versions as compared to the brand product. Of note, two of the five generic forms tested had extremely fast onset of disintegration (2-3 seconds), which was not observed with the brand versions. The disintegration times of generic risedronate from the Walker and Adachi [22] trial were longer than the bran, whereas the results of this trial found generic alendronate tablets to disintegrate far faster than brand.

Since there are differences in disintegration times and differences in the excipients between brand and generic forms of alendronate, it is reasonable to assume that there are differences in when alendronate is made available to the body for absorption. Shakweh et al. [21] reported significantly (*P* < 0.05) greater esophageal bioadhesion with some generic forms of alendronate as compared to brand alendronate and postulated that the differences in the bioadhesive characteristics were likely due to the differing inactive ingredients between the brand and generic forms. Further, several generic tablets tested in this trial displayed cleavage rupture in the oesophagus, increasing the probability of adhesion of pieces of the tablet to the oesophageal wall, increasing the probability of irritation. Perkins et al. [14] assessed the oesophageal transit time of branded risedronate and two generic alendronate formulations. A semisitting posture, as opposed to being upright, significantly slowed esophageal transit. Further, generic alendronate formulations had significantly (*P* < 0.05) slower transit times than risedronate. This significantly (*P* < 0.05) slower transit time may have been a consequence of the higher bioadhesion characteristics with some generic forms of alendronate as reported in Shakweh et al. [21].

It is well established that there is poor persistence and compliance to bisphosphonate therapy in general [23, 24]. Two investigations of administrative databases for the province of Quebec, Canada, compared persistence to generic alendronate to that of either brand risedronate or brand alendronate [25, 26]. Of note, in Quebec all medication expenses are reimbursed by the provincial formulary, removing one of the greatest barriers to drug adherence—cost. In one of the two investigations, after adjusting for demographic differences among the groups, there was an approximate two-fold greater risk of discontinuation of therapy for those patients who initiated with generic alendronate as compared to those with a brand bisphosphonate (hazard ratio = 2.08; 95% confidence interval 1.89–2.28) [25]. In the other study, significantly (*P* < 0.05) greater discontinuation of therapy was reported with generic alendronate compared to brand bisphosphonates in patients prescribed bisphosphonates for either primary or secondary prevention [25]. The reasons behind the lower persistence with generic alendronate as compared to brand alendronate in both of these trials is unknown but cannot be attributed to differences in patient costs.

A chart review from two specialized tertiary care referral centers in Canada was undertaken to quantify changes in adverse-event rates, changes in bone mineral density, and discontinuation among postmenopausal women greater than 50 years of age before and after switch from brand to generic alendronate [27]. Patients who were previously stable on doses of brand alendronate experienced an increase in adverse events causing discontinuation after the introduction of widespread automatic substitution to generic alendronate. Of note, adverse events were severe enough to warrant discontinuation in 21% of cases using brand alendronate and in 79% of cases with generic alendronate. In addition, significant (*P* < 0.05) reductions in lumbar spine and femoral neck bone mineral density were recorded in patients given generic alendronate despite having previously experienced stable bone mineral density while being on brand alendronate. Lastly, three contemporaneous cohorts (weekly brand alendronate, brand risedronate, or generic alendronate) were retrospectively studied in chart review of 186 women from a German tertiary clinic [28]. The investigators reported that women provided generic alendronate had significantly (*P* < 0.05) more gastrointestinal adverse events (15, 9, and 32, resp.), significantly (*P* < 0.05) smaller gains bone mineral density at both the lumbar spine (5.2, 4.8, and 2.8%, resp.) and total hip (2.9, 3.1, and 1.5%, resp.), and significantly (*P* < 0.05) lower persistence after 12 months (84, 94, and 68%, resp.) when compared to women given either of the brand bisphosphonates. The authors postulated that the smaller gains in bone mineral density (40–50% lower) with the generic could be a factor of lower persistence and/or of lower bioavailability or potency of the generic alendronate. These two chart review are consistent in their findings of differences between brand and generic bisphosphonates. While retrospective cohort data is subject to some bias in that there is generally a lack of blinding, it provides further evidence of differences between brand and generic alendronate with respect to tolerability and effectiveness.

There were a few limitations to our investigation. When the tablet disintegration time was assessed there was no blinding as to tablet type. Also, differences in* in vitro* tablet disintegration do not necessarily translate into differences in* in vivo* tablet dissolution.

## 5. Conclusions

Canadian-based generic formulations of alendronate had significantly (*P* < 0.05) faster disintegration times as compared to brand alendronate or risedronate. The rapid disintegrations of the generic formulations assessed were found to be similar to those reported for tablets specifically designed to disintegrate in the mouth (<30 seconds). These significant differences in disintegration times between generic and brand bisphosphonates may explain some of the differences that are reported concerning the inferior effectiveness, safety, and persistence to generic bisphosphonates as compared to their respective brands.

## Figures and Tables

**Table 1:** Amino-bisphosphonate tablet group sample size, lot numbers, and disintegration times.

Tablet and dose	Lot numbers	Mean disintegration time in seconds (SD^a^)	Number of tablets tested
Novo-alendronate 70 mg	A34021	12.7 (1.09)	18
Apo-alendronate 70 mg	(L) JD 7416	25.7 (5.59)	20
Brand risedronate 35 mg	425314	101.2 (20.56)	20
Brand alendronate 70 mg plus vitamin D 5600 IU	Y 1382	378.0 (60.5)	20
Brand alendronate 70 mg	Y1277 and Y1498	147.4 (50.47)	20

^a^SD: standard deviation.

**Table 2:** Comparisons of mean disintegration times among brand and generic bisphosphonates.

Bisphosphonate tablet comparison	Mean disintegration time difference in seconds	95% CI^a^
Brand alendronate—brand alendronate plus vitamin D	−231∗	264, 197
Brand alendronate—risedronate	46∗	13, 80
Brand alendronate—Apo-alendronate	122∗	88, 155
Brand alendronate—Novo-alendronate	135∗	100, 169
Brand alendronate plus vitamin D—risedronate	277∗	243, 310
Brand alendronate plus vitamin D—Apo-alendronate	352∗	319, 386
Brand alendronate plus vitamin D—Novo-alendronate	365∗	331, 400
Risedronate—Apo-alendronate	76∗	42, 109
Risedronate—Novo-alendronate	89∗	54, 123
Apo-alendronate—Novo-alendronate	13	−20, 46

^a^95% CI = 95% confidence interval; *significant disintegration time difference between groups at *P* < 0.05.
